# Bacteremia in Patients with Solid Organ Cancer: Insights into Epidemiology and Antibiotic Consumption

**DOI:** 10.3390/cancers15235561

**Published:** 2023-11-24

**Authors:** Begoña de Dios-García, Guillermo Maestro, Carmen Díaz-Pedroche, Wagner Parra, Óscar Campos, María Ángeles Orellana, José Manuel Caro, Carlos Lumbreras, Manuel Lizasoain

**Affiliations:** 1 Internal Medicine Department, Universitary Hospital “12 de Octubre”, Av Córdoba, 28041 Madrid, Spain; guillermo.maestro@salud.madrid.org (G.M.); cdiazp@salud.madrid.org (C.D.-P.); carlos.lumbreras@salud.madrid.org (C.L.); manuel.lizasoain@salud.madrid.org (M.L.); 2 Oncology Department, Universitary Hospital “12 de Octubre”, Av Córdoba, 28041 Madrid, Spain; wagnergonzalo.parra@salud.madrid.org (W.P.); oscar.campos@salud.madrid.org (Ó.C.); 3 Microbiology Department, Universitary Hospital “12 de Octubre”, Av Córdoba, 28041 Madrid, Spain; mariaangeles.orellana@salud.madrid.org; 4 Pharmacy Department, Universitary Hospital “12 de Octubre”, Av Córdoba, 28041 Madrid, Spain; josemanuel.caro@salud.madrid.org

**Keywords:** solid organ cancer patients, bacterial resistance, antibiotic consumption, hospitalist care, hospital co-management

## Abstract

**Simple Summary:**

Prior knowledge of local epidemiology is useful to optimize antibiotic prescription. Solid organ cancer patients (SOC) are frequently prescribed antibiotics, as we confirmed in our retrospective study. Our aim was to describe risk factors for bacterial resistance in SOC patients with bacteremia in order to determine the adequacy of antibiotic treatment, improve outcomes, and diminish the emergence of bacterial resistance. We compared the rates of bacterial resistance in oncology and medical wards, as well as antibiotic consumption. We observed that resistance rates in worrisome bacteria were not higher in the oncology ward than in medical wards. Furthermore, consumption of broad-spectrum antibiotics was reduced, in which hospital co-management with the internal medicine department may have played a role. The presence of a urinary catheter and previous antibiotic consumption were risk factors for bacterial resistance.

**Abstract:**

Epidemiology and risk factors associated to bacterial resistance in solid organ cancer (SOC) patients has been barely described. This retrospective monocentric study analyzed clinical variables in SOC patients who developed bacteremia between 1 January 2019 and 31 December 2022. We described rates of bacterial resistance in Gram negative bacteria (80.6%): *E. coli-ESBL, K. pneumoniae-ESBL*, Carbapenem-Resistant *K. pneumoniae* and Meropenem-Resistant *P. aeruginosa,* as well as antibiotic consumption, and compared these rates between the medical and oncology wards. In total, we included 314 bacteremias from 253 patients. SOC patients are frequently prescribed antibiotics (40.8%), mainly fluoroquinolones. Nosocomial bacteremia accounted for 18.2% of the cases and only 14.3% of patients were neutropenic. Hepatobiliary tract was the most frequent tumor (31.5%) and source of bacteremia (38.5%). Resistant bacteria showed a decreased rate of resistance during the years studied in the oncology ward. Both K-ESBL and K-CBP resistance rates decreased (from 45.8% to 20.0%, and from 29.2% to 20.0%, respectively), as well as MRPA, which varied from a resistance rate of 28% to 16.7%. The presence of a urinary catheter (*p* < 0.001) and previous antibiotic prescription (*p* = 0.002) were risk factors for bacterial resistance. Identifying either of these risk factors could help in guiding antibiotic prescription for SOC patients.

## 1. Introduction

Antimicrobial resistance is one of the threats we face when treating patients with infections. In recent years, antibiotic consumption has increased and bacterial resistance has spread worldwide, and has become a global health problem requiring active surveillance according to the European Centre for Disease Prevention and Control [1]. Notably, carbapenem-resistant Enterobacterales represent a common warning due to the few efficient treatments available. A higher mortality has been described when compared to carbapenem-susceptible bacteria, mostly studied with *K. pneumoniae* [2], still considered a nosocomial bacteria.

Cancer patients may be exposed to an increased risk of healthcare or nosocomial infections by resistant bacteria due to their frequent use in hospital facilities. Bacteremic infections pose a life-threatening risk to cancer patients for which prompt and adequate treatment must be prescribed. In the era of multiresistant bacteria, it is mandatory to be well informed of the local epidemiology in order to guarantee a successful treatment.

More than a decade ago, the epidemiology of bacteremia in cancer patients was described mostly among oncohematological patients, and specifically in neutropenic patients [3,4]. 

Subsequently, different studies confirmed the change in the epidemiology from Gram-positive bacteria to Gram-negative bacteria as the leading cause of bacteremia in cancer patients [5]. Several reasons for this were described: (1) the use of non-myeloablative regimens with less profound neutropenia and less severe mucositis, (2) the increasing placement of peripheral central line catheters, and (3) the withdrawal of fluoroquinolone prophylaxis. Again, these studies included mainly patients with hematological malignancies.

In more recent years, the treatment for solid organ cancer (SOC) patients has changed enormously due to immune-modulatory therapies. Thus, the risk factors and epidemiology of bacteremia in these patients have been barely described.

A Spanish group published studies focusing on the outcome and prognosis in SOC patients [6,7]. In 2011 they also described risk factors for multidrug-resistant bacteria (MDR), but this study only included 23 SOC patients [8]. It was also reported that MDR might aggravate the prognosis of SOC patients due to the delay in the prescription of adequate antibiotics. A few years later, the same group drew attention to the increasing emergence of antibiotic resistance in these patients [9], but carbapenemase-producing bacteria were not a common problem at that time. Altogether, there are no recent data regarding bacteremia among SOC patients.

Precise knowledge of local epidemiology contributes to the optimization of antibiotic treatment and improves the outcome of cancer patients who are at increased risk of developing fatal consequences after an infection, due to immunosuppression. Although bacterial resistance has been linked to immunosuppression, it is essential to identify the risk factors associated with this resistance in order to provide a proportional and adequate use of wide-spectrum antibiotics. The spread of MDR has become a major health problem worldwide [10] and efforts are focused on Antimicrobial Stewardship Programs (ASP) to reduce antibiotic misuse and overuse [11]. In Spain there exists a National Strategic Plan on Antimicrobial Resistance [12]. 

Hospitalist care in oncology units is being implemented with encouraging results [13,14]. Internal medicine physicians display a predisposition to implement ASP and may contribute positively to an adequate level of antibiotic prescription.

The objective of this study was to analyze the possible variables associated with bacterial resistance in bacteremia in SOC, so that this aggravating condition may be prevented or appropriately addressed.

## 2. Materials and Methods

A descriptive retrospective study was conducted at the Hospital Universitario “12 de Octubre” (Madrid, Spain) among adult patients diagnosed with SOC and with one or more episodes of bacteremia between 1 January 2019 and 31 December 2022. The following variables were collected: age, sex, type of tumor, source of bacteremia, presence of catheter (central line, urinary or biliary), chronic steroid treatment, antibiotic treatment in the preceding three months (ATB3m) and development of febrile neutropenia. The source of infection was defined as the infective focus possibly responsible for the bloodstream infection, based on clinical history, physical examination, and complementary results revised by an oncologist or an internal medicine physician. 

Bacteremia was considered nosocomially acquired if it appeared more than 48 h after admission. Neutropenia was defined as an absolute neutrophil count of <500 cells/mm^3^. Corticosteroid therapy was considered when the patient was taking corticosteroids at the time of bacteremia onset.

Patients were identified using blood culture data from the microbiology laboratory information system. Bacteremia was defined by the growth of defined bacteria in one or more blood culture bottles and identified as significant by the corresponding clinician. Two sets of blood samples were taken, when patients presented fever ≥37.8 °C or when bacteremia was suspected clinically. Blood samples were inoculated in BacT/Alert^®^ FA aerobic and FN anaerobic bottles and incubated in a BacT/Alert^®^ Virtuo system (bioMérieux, Marcy-l’Etoile, France) for 5 days. Following a positive signal, Gram staining and subculturing in solid growth media, including blood, chocolate and Schaedler agar plates (bioMérieux, Marcy-l’Etoile, France), were performed. The identification and sensitivity studies were performed with matrix-assisted laser desorption/ionization time-of-flight (MALDI-TOF) mass spectrometry (Bruker Daltonics, Billerica, MA, USA) and the MicroScan WalkAway^®^ system (Beckman Coulter, Brea, CA, USA). The criteria of The European Committee on Antimicrobial Susceptibility Testing (EUCAST) for susceptibility methods were used to define antibiotic resistance.

Bacterial resistance was analyzed according to the presence of: an Extended-Spectrum-Betalactam in the case of *E. coli*-ESBL (E-ESBL) and *K. pneumoniae* (K-ESBL), *K. pneumoniae* Carbapenem-Resistant (K-CBP), *E. cloacae* Carbapenem-Resistant (E-CBP), *P. aeruginosa* Meropenem-Resistant (MRPA), *S. aureus* Methicillin-Resistant (MRSA) and *E. faecium* Vancomycin-Resistant (VREf). Multidrug-resistant bacteria were considered for Gram-negative bacteria (GNB) and included Extended-Spectrum Betalactam or carbapenem-resistant isolate. 

Our Oncology Ward includes a co-management team composed of oncologists and internal medicine physicians. The role of the internal medicine physician is defined as hospitalist care in our study.

Our institution has an ASP that reports annually the rates of antibiotic resistance of these bacteria that might pose difficulties for adequate antibiotic treatment. The rates of bacterial resistance in the oncology ward were compared to global medical wards. 

To analyze factors associated with antibiotic resistance, we analyzed the consumption of antibiotics and compared the Oncology Ward (OW) with the Medical Ward (MW). We compared defined daily doses (DDD)/100 admissions in both wards.

We performed a univariate analysis of variables associated with antibiotic resistance using Chi-square tests and Student *t*-tests for categorical and continuous variables, respectively. Odds ratios (ORs) and 95% confidence intervals (CIs) were calculated. We then performed multivariate conditional logistic regression analyses including variables with a *p*-value of ≤0.10 and clinically significant variables. The analyses were performed using the stepwise Logistic Regression model in SPSS Statistics, Version 24.0 (SPSS Institute Inc., Chicago, IL, USA). 

## 3. Results

During the period considered in this study, there were 314 bacteremias in SOC patients, corresponding to 303 episodes in 253 patients. The yearly distribution of bacteremias was as follows: 90 in 2019 (28.7%), 72 in 2020 (22.9%), 87 in 2021 (27.7%), and 65 in 2022 (20.7%). 

The characteristics of the patients studied are shown in Table 1. The type of tumor more frequently associated with bacteremia was of hepatobiliary origin (31.5%), followed by those of gynecologic and urinary origin (13.1% for both types). 

Over the three months prior to their admission to the hospital, 40.8% of patients had received one or more antibiotics with the following distribution: Fluoroquinolones (33.6%), Third-generation Cephalosporines (29.7%), Carbapenems (25.8%), Amoxicilin/Clavulanic Acid (24.2%), and Piperacilin/Tazobactam (21.1%) (Table 2). 

The most frequent source of bacteremia was of biliary origin, 121 cases (38.5%); followed by the urinary tract, 83 cases (26.4%); and abdominal site, 40 cases (12.7%). The presence of a catheter was common in cases with an endovascular (89.5%), biliary (56.8%), or urinary (41.0%) source. Nosocomial bacteremia represented 18.2%. Only 14.3% of bacteremic patients were neutropenic (Table 1).

The types of bacteria isolated each year are represented in Table 3. There were 253 bacteremias caused by GNB (80.6%). The most frequent bacteria found were: *E. coli*, 147 cases (46.8%) (E-ESBL 15.6%); *K. pneumoniae*, 70 cases (22.3%) (K-ESBL 29.6%; K-CBP 22.5%); *P. aeruginosa*, 29 cases (9.2%) (*P. aeruginosa* Meropenem-Resistant (MRPA) (28.6%); and *E. cloacae* CBP-R 7 cases (2.2%). 

Gram-positive bacteria (19.4%) were represented by: 35 cases of *S. aureus* (56.5%, 7.4% *S. aureus* Methicillin Resistant (MRSA)); 12 cases of *S. pneumoniae* (19.4%); and 15 cases of *E. faecium* (24.2%, Enterococcus-VanR 9.1%). Globally, MDR-GNB represented 26.9% of the total isolates.

We observed that antibiotic resistance varied during the years studied. Table 4 shows the rates of bacterial resistance in the OW and the MW. *E. coli*-ESBL increased from 13.2% (2019) to 16.1% (2022), similar to the global hospital resistance rate. Both the K-ESBL and the K-CBP resistance rates decreased (from 45.8% to 20.0%, and 29.2% to 20.0%, respectively) while their global hospital resistance rates increased. MRPA varied from a resistance rate of 28% to 16.7%, similar to that observed in medical wards.

Factors associated with bacterial resistance were as follows: ATB3m (62.5% vs. 36.5%; *p* ≤ 0.001), presence of a urinary catheter (28.6% vs. 10.1%; *p* < 0.001), and development of a nosocomial bacteremia (28.6% vs. 15.9%; *p* = 0.03). The multivariate analysis yielded that the use of antibiotics in the previous 3 months [2.69 (1.45–4.98); *p* = 0.002] and the presence of a urinary catheter [3.43(1.65–7.14); *p* < 0.001] were risk factors for bacterial resistance (Table 5). The absence of any of these factors prevented bacterial resistance [0.24 (0.12–0.49); *p* < 0.001].

**Figure 1:**
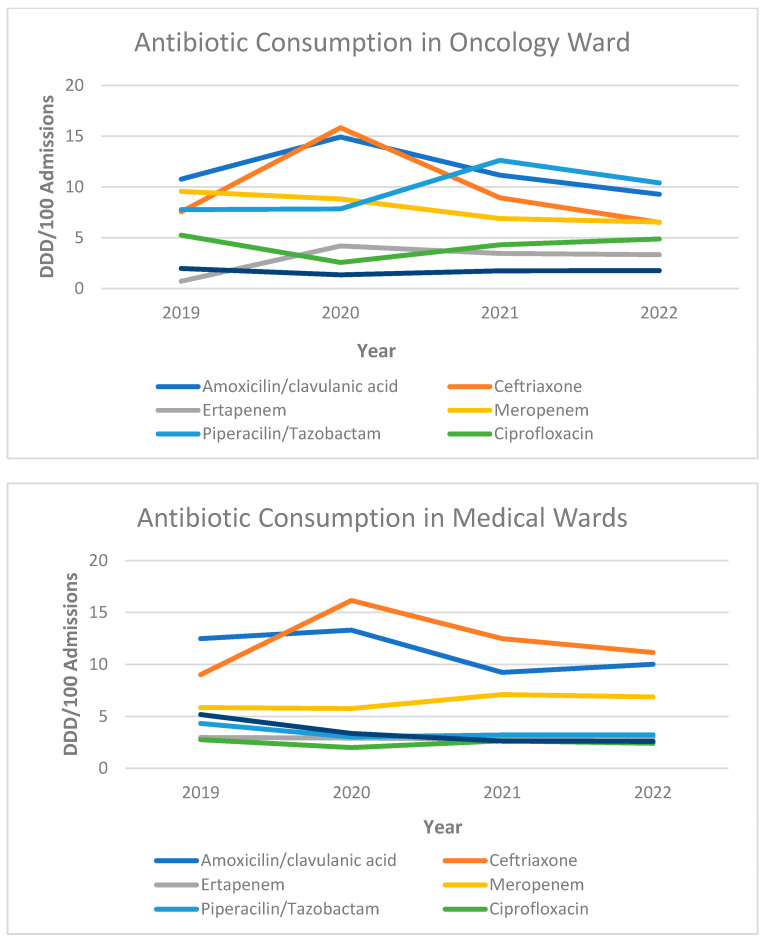
Antibiotic consumption.

Antibiotic consumption differed between the two wards (Figure 1). We observed a reduction in prescription of meropenem running parallel to an increase in prescription of piperacillin/tazobactam in the OW. However, fluoroquinolone consumption showed an inverted tendency: ciprofloxacin was highly prescribed in the OW while it was hardly prescribed at all in the MW. Levofloxacin consumption was steady in the OW while it decreased in the MW.

## 4. Discussion

A main objective of this study was to provide new evidence for the epidemiology and characteristics of bacteremia in SOC patients in a hospital with a high percentage of oncologic admissions, thus expanding previous studies published more than a decade ago. Indeed, bacterial resistance in these patients has received limited attention. A previous study by a Spanish group [8] explored risk factors for being infected by MDR bacteria and mostly included patients with hematologic malignancies (HM). One of our aims was to focus exclusively on SOC patients, as the consequent immunosuppression and treatment of their disease strongly differs from HM patients.

Contrary to what was described in HM patients, our study shows that bacteremia is not associated with febrile neutropenia in SOC patients. Bacteremia developed in non-neutropenic patients in 86% of the cases, in agreement with previous reports [15,16]. Recent treatments based on immune-modulatory therapies may produce less damage to anatomical barriers, such as mucous membranes, thus preventing the development of bacterial invasion. Furthermore, when SOC patients develop neutropenia they seldom present dysfunctional neutrophils, as nowadays therapeutic schemes are less myeloablative. Indeed, duration of neutropenia rarely last longer than 7 days. Thus, SOC patients are considered to be low-risk neutropenia patients, even allowing outpatient treatment. Therefore, neutropenia does not seem to entail a higher risk for a worse prognosis in SOC patients.

We also found that 18% of bacteremia was of nosocomial origin, a percentage which is similar to that described by Anatoliotaki et al. [15] and Marín et al. [6]. Several factors may contribute to this result, such as the use of catheters, which are usually placed and changed during hospitalization. The manipulation and handling of catheters are known to be risk factors for bacteremia. It is interesting that most of the infections in SOC patients would probably have to be considered health-related infections, as these patients come frequently to the hospital either for treatment administration or catheter manipulation. This fact could have increased the rate of nosocomial bacteremia.

In our study, the type of tumor and the source of bacteremia were more frequently in the hepatobiliary tract. However, Anatoliotaki et al. described that obstructive phenomena constitute a predisposing condition to the development of infection in SOC patients. A previous study by Marín [6] also described similar findings. These results are not surprising for several reasons. Firstly, the liver is a common metastatic site for many tumors and changes in liver anatomy would certainly favor either invasion of the biliary tract or an increase in intrabiliary pressure, both favoring bacteremia. Secondly, the liver receives, via the portal vein, blood flow from the lower intestinal tract where a high density of bacteria exists. Thirdly, the biliary tract is a highly bacteremic source, even in non-cancer patients. 

A noteworthy fact is that urinary tract bacteremia is frequently related to the presence of a catheter. Indeed, the frequency of urinary tract obstruction in urinary and gynecologic tumors requires the placement of a catheter, which supports its high incidence source.

Of the patients studied, 40.8% had previously been prescribed antibiotics. Consumption of antibiotics has been related to the emergence of bacterial resistance [17]. SOC patients have factors that may increase their risk for being prescribed antibiotics: a higher frequency of infections as febrile neutropenia, mucositis, the presence of catheters or an impaired immunity. Due to a higher frequency of antibiotic prescription we would expect a higher antibiotic resistance, as it is observed with specific worrisome bacteria, such as *K. pneumoniae-ESBL* and *P. aeruginosa*. Global betalactam resistance is progressively increasing, including at our institution. The first multicenter study focusing on risk factors for *E. coli*-ESBL in Spain [18] showed that healthcare-associated bacteremia, urinary catheter use, and antimicrobial prescription in the previous 2 months were associated with bacterial resistance. Specifically, fluoroquinolones and cephalosporins were the most frequently associated antibiotics, in agreement with our present results. 

We found an elevated MDR-GNB incidence (26.9%) compared to previous studies [8]. We should take into account that during the first wave of the SARS-CoV2 pandemic, cephalosporins were commonly prescribed and they have been associated with the emergence of ESBL bacteria in bacteremic patients with hematologic malignancies [19]. Furthermore, fluoroquinolones were the most commonly prescribed antibiotics in SOC patients in our series. The reasons for this could be that fluoroquinolones are the indicated antibiotics for oral treatment in low-risk febrile neutropenia and their high bioavailability favors their use in oral switch. Ciprofloxacin prophylaxis in hematologic malignancies has been abandoned for long neutropenic patients, a fact that has influenced changes in their epidemiology [20], but prescription of ciprofloxacin in SOC patients continues to be high.

With the emergence of carbapenem-resistant bacteria several risk factors have been identified. These include previous antibiotic exposure to cephalosporins, fluoroquinolones, and carbapenems; prior colonization; admission to an intensive care unit; and an extended hospital stay [21]. These results highlight that the development of a CBP-resistant bacterial infection is mainly nosocomial, and the use of empirical antibiotics may be broadened in these cases.

As antibiotic consumption runs parallel to the emergence of resistance, by showing the antibiotic consumption during the years of the study we wanted to point out some observations: *E. coli*-ESBL is steady increasing, as it is in the community setting, so the reasons for this increase might be found outside the hospital setting. *K. pneumoniae* infection represents a world global threat and previous studies describing carbapenem-resistant bacteria were focused on this species [22]. However, *K. pneumoniae* (*K*-ESBL and *K*-CBP) as an emerging resistant species, had a reduced incidence rate in the oncology ward. In parallel, we see that ceftriaxone and meropenem have been prescribed less, and we hypothesize that a correlation might exist between the two facts. 

Interestingly, even when the prescription of antibiotics in SOC patients was high, we observed a decrease in the percentage of resistance. One explanation for this could be the co-management between the oncology ward and the internal medicine department. It is difficult to attribute our results to the co-management intervention as our study was not designed for this purpose. Hospitalist care might enhance the implementation of antimicrobial stewardship programs as hospitalists may be more familiar with it, while oncologist care may be more concentrated on the tumor aspects. This fact might have favored the choice of antibiotic according to local guidelines, shortened the duration of antibiotic treatments, and promoted an early oral switch and de-escalation, minimizing antibiotic resistance emergence. In agreement with this, the role of the hospitalist in improving the implementation of effective measures to control infectious diseases has been previously described [13]. 

This is clearly seen when looking at meropenem prescription in the OW which gradually decreased, favoring piperacillin/tazobactam prescription, which implies active promotion of a carbapenem-sparing regimen. Published studies comparing hospitalist care and oncology have focused on prognosis and cost, but to our knowledge, this is the first study showing that internal medicine co-management could be beneficial in minimizing antibiotic pressure.

An important finding of this study is that are there are basically two factors associated with bacterial resistance: previous use of antibiotics and the presence of a urinary catheter. The identification of either of these risk factors in our SOC patients could represent a sign towards a wide-spectrum antibiotics prescription. Or, on the contrary and even more important, the absence of these factors may limit the need for such a prescription.

Several limitations of our study are worth mentioning. First, this is a single-centered and retrospective study, which makes it difficult to establish comparisons. Although, we acknowledge its limitations, we consider that describing local epidemiology will encourage other groups to upgrade and publish their findings, broadening information about the epidemiology of bacterial resistance in SOC patients which is currently lacking. Second, the stage and type of treatment of the oncologic disease was not recorded. This fact would have helped in distinguishing different grades of immunosuppression. Third, we point out that manipulation is a risk factor for bacteremia, but invasive procedures were not recorded. However, although we did not analyze the appropriateness of antibiotic treatment our mortality rate was low, which reinforces that knowledge of local epidemiology and compliance with our local antibiotic protocol is mandatory to ensure a good prognosis. It is interesting that most of the infections in SOC patients would probably have to be considered health-related, as the patients come frequently to the hospital for treatment administration or catheter manipulation. This factor could have increased the rate of nosocomial bacteremia. 

Finally, we would like to mention that, although we did not evaluate the mortality risk factors, corticosteroids are increasingly used in SOC patients, particularly in high doses for those with immune-mediated complications. We would like to focus attention on this fact, which could represent a potential risk factor for bacterial resistance in the near future.

The main risk factors for bacterial resistance in our study were the previous consumption of antibiotics in the preceding three months and the presence of a urinary catheter. The recognition of either of these factors would help in guiding antibiotic prescription. Moreover, hospital co-management between internal medicine and oncology departments may promote adequate levels of antibiotic treatment prescription and prevent bacterial resistance emergence.

## 5. Conclusions

The main risk factors for bacterial resistance in our study were the presence of a urinary catheter, the nosocomial origin of bacteremia, and the previous consumption of antibiotics in the preceding three months. The recognition of any of these factors would help in guiding antibiotic prescription. Moreover, hospital co-management between the internal medicine and oncology departments may promote adequacy of antibiotic treatment prescription and prevent bacterial resistance emergence.

## Figures and Tables

**Table 1:** Clinical characteristics of bacteremic SOC patients.

	*n* = 253
Age, median (range)	67 (58–73)
Sex (men/women)	149/104
Type of tumor (%)	
Breast	22 (7.0)
Colon	30 (9.6)
Germinal	3 (1.0)
Gynecologic	41 (13.1)
Head and neck	6 (1.9)
Hepatobiliary	99 (31.5)
Lung	33 (10.5)
Melanoma	3 (1.0)
Neuroendocrine	13 (4.1)
Upper gastrointestinal tract	5 (1.6)
Urinary tract	41 (13.1)
Other	13 (4.1)
Nosocomial (%)	57 (18.2)
Type of catheter (%)	
Biliary	73 (23.2)
Central line	78 (24.8)
Urinary	42 (13.4)
Previous antibiotic within 3 m (%)	128 (40.8)
Febrile Neutropenia (%)	45 (14.3))
Corticosteroids (%)	86 (27.4)
Bacteremia source (%)	
Abdominal	40 (12.7)
Biliary tract	122 (38.5)
Central nervous system	3 (1.0)
Endovascular	20 (6.1)
Osteoarticular	1 (0.3)
Primary	11 (3.5)
Respiratory	26 (8.3)
Skin and soft tissue infection	8 (2.5)
Urinary	83 (26.4)
Mortality (%)	32 (10.2)

**Table 2:** Antibiotic prescription in the previous 3 months.

Betalactams	%
Third-generation cephalosporin	381 (29.7)
Piperacilin/tazobactam	27 (21.1)
Amoxicilin/clavulanic acid	31 (24.2)
Carbapenem	33 (25.8)
Other betalactam	20 (15.6)
Fluoroquinolones	43 (33.6)
Other antibiotic	43 (33.6)

Percentage referred to mean.

**Table 3:** Types of bacteria per year.

Bacteria/Year	2019 *n* = 90	2020 *n* = 72	2021 *n* = 87	2022 *n* = 65	2019–2022 *n* = 314
*E. coli*	38 (42.2)	42 (58.3)	36 (41.4)	31 (47.7)	147 (46.8)
*K. pneumoniae*	23 (26.1)	12 (16.7)	20 (23.0)	15 (23.1)	70 (22.3)
*E. cloacae*	3 (3.4)	2 (2.8)	1 (1.1)	1 (1.5)	7 (2.2)
*P. aeruginosa*	7 (8.0)	8 (11.1)	7 (8.0)	6 (9.2)	29 (9.2)
*S. aureus MR*	11 (12.5)	7 (9.7)	9 (10.3)	8 (12.3)	35 (11.1)
*E. faecium*	-	-	12 (13.8)	3 (4.6)	15 (4.7)
*S. pneumoniae*	7 (9.1)	1 (1.4)	2 (2.4)	1 (1.5)	11 (3.5)

Results are expressed in %.

**Table 4:** Bacterial resistance in Gram-negative bacteria.

Year	2019		2020		2021		2022		2019–2022 *	
Bacteria	MW	OW	MW	OW	MW	OW	MW	OW	MW	OW
*E. coli* ESBL	15.6	13.2	14.1	11.9	18.7	22.2	20.2	16.1	17.1	15.6
*K. pneumoniae* ESBL	20.7	45.8	17.3	25.0	16.3	20.0	15.3	20.0	17.5	29.6
*K. pneumoniae* CBP-R	17.1	29.2	24.0	16.7	21.1	20.0	15.3	20.0	18.8	23.9
*E. cloacae* CBP-R										
*P. aeruginosa* Meropenem-R	29.3	28.6	24.0	37.5	16.7	28.6	14.0	16.7	20.5	28.6

* Mean; MW: medical ward; OW: oncology ward.

**Table 5:** Analysis of risk factors for resistant bacteria.

Chi-Square-Test					Univariate Analysis		Multivariate Analysis		
	BacRes	Non-BacRes	*p* Value	OR	IC 95%	*p* Value	OR	IC 95%	*p* Value
Central line	17.9	26.5	0.18						
Urinary catheter	28.6	10.1	<0.001	3.57	1.76–7.24	0.001	3.43	1.65–7.14	0.001
Biliary catheter	25.0	23.0	0.75						
Previous antibiotic 3m	62.5	36.5	<0.001	2.90	1.60–5.28	<0.001	2.69	1.45–4.98	0.002
Febrile neutropenia	7.3	16.0	0.14						
Corticosteroids	32.7	26.6	0.35						
Nosocomial bacteremia	28.6	15.9	0.02	2.11	1.08–4.13	0.03			

## Data Availability

Data are unavailable due to privacy or ethical restrictions.
